# Total systems failure: police officers’ perspectives on the impacts of the justice, health, and social service systems on people who use drugs

**DOI:** 10.1186/s12954-022-00629-1

**Published:** 2022-05-19

**Authors:** Amanda Butler, Naomi Zakimi, Alissa Greer

**Affiliations:** School of Criminology, Simon Fraser University, 8888 University Drive, Burnaby, BC V5A 1S6 Canada

## Abstract

**Background:**

Police in Canada have become main responders to behavioural health concerns in the community—a role that disproportionately harms people who use drugs (PWUD). Recent calls to defund the police emphasize the need to shift responsibility for non-criminal health issues from police to health and social services. This study explores the role of police interactions in responding to PWUD within the broader institutional and structural contexts in which they operate.

**Methods:**

We conducted a qualitative thematic analysis of interviews with sixteen police officers across nine jurisdictions in British Columbia, Canada. We examined police officers’ everyday policing experiences interacting with PWUD, enforcing drug laws, and working alongside other service sectors.

**Results:**

Officers explained that the criminal justice system is one component of a wider network of systems that *collectively* fail to meet the needs of PWUD. They recognized that PWUD who interact with police often experienced intersecting structural vulnerabilities such as poverty, homelessness, and intergenerational trauma. Harmful drug laws in conjunction with inadequate treatment and housing resources contributed to a funnelling of PWUD into interactions with police. They provided several recommendations for reform including specialized health and justice roles, formalized intersectoral collaboration, and poverty reduction.

**Conclusions:**

Overall, this study provides unique insights into the positioning and role of police officers within a “total systems failure” that negatively impact PWUD. Police have become responders-by-default for issues that are fundamentally related to people’s health conditions and socioeconomic circumstances. Addressing failures across the health, social, and justice systems to meet the needs of PWUD will require an examination of the shortcomings across these systems, as well as substantial funding and system reforms.

## Background

An estimated $100 billion worldwide is spent annually on law enforcement-led approaches to combating drugs [1]. The “War on Drugs” is associated with increased drug toxicity, exacerbation of health and social inequities, stigma, mass incarceration, human rights violations, and increased transmission of blood-borne diseases [2, 3]. This punitive approach to drug use can be aptly described as “an expensive way to make a bad problem worse” [4]. International illicit drug markets are expanding and becoming more dangerous in spite of global drug prohibition [5]. The World Health Organization [6] and global leaders increasingly agree that drug prohibition has failed comprehensively, leading some countries to consider alternative drug policy approaches, including depenalization, decriminalization, and legalization. While it may be an attractive political strategy to present the harms associated with drug dependence as a *consequence* of illicit drug use, studies suggest that chronic problematic drug use is more often a *symptom* of wider underlying issues, not the causal factor [7]. For example, lifetime and childhood trauma are among the best predictors of substance use disorder [8].

Police officers frequently encounter people with mental health and substance use issues—a subgroup of people who often experience complex health and social needs. Some police agencies in Canada have publicly recognized their role in responding to people experiencing mental health and substance use-related crises and have committed to improving outcomes through education, training and partnerships [9]. For example, the Vancouver Police Department released the *Vancouver Police Mental Health Strategy* in 2016, wherein it acknowledged the importance of diversion, education, systematic stigma reduction, and collaborating upstream with health care providers to better serve people with mental illness and/or substance use needs [10]. However, the extent to which this role is recognized, resourced and accepted is inconsistent across the country as police services, which operate at all levels of government in Canada, have distinct mandates and accountability structures. Most large Canadian cities, such as Vancouver, maintain their own municipal police force. In contrast, Royal Canadian Mounted Police (RCMP), the federal and national police service of Canada, cover suburban and urban cities along with several remote and rural jurisdictions.

As the first point of contact, police officers may play an important role in early intervention and preventing advancement into the criminal justice system [11]. Police are generally encouraged to divert people with complex health-related needs away from the criminal justice system by connecting them to treatment and other behavioural health services. But the success of criminal justice diversion practices depend on the availability of community-based resources for police to divert *to* [12] and on collaboration with public health organizations. Insufficient resources available for people who use drugs (PWUD) may be linked to frequent police encounters among this population [13]. We need to critically consider the potential consequences of an expanded scope for police in health and harm reduction spaces given that police presence may exacerbate harms among PWUD. For example, studies suggest that police surveillance limits access to harm reduction services thereby increasing involvement in risky behaviours such a syringe sharing [13–15] and PWUD report that fear of police as a barrier to calling emergency medical services in the event of an overdose [16]. Furthermore, interactions between PWUD and police, and the impacts of these interactions, are shaped by the social conditions in which people live.

### How social conditions shape police interactions and outcomes for PWUD

Beyond simple drug possession, PWUD may be involved in other activities that increase their likelihood of interacting with police such as property crime, trafficking, violence, and sex work [17, 18]. Discussions about the drug–crime relationship often fail to consider the intersectional structural vulnerability of PWUD who face multiple, overlapping inequities that contribute to the ongoing harms that they experience [19]. For instance, there is a well-established synergistic relationship between drug use and poverty [20], which is also related to frequent police encounters [13]. PWUD often belong to multiple intersecting socially excluded groups, and they face myriad obstacles to accessing health and social services. PWUD experience stigma, housing unaffordability, poor therapeutic relationships, and lack of trust in the health care system [21, 22] to a greater extent that those who do not use drugs. A study of opioid overdoses in 2016–2017 found that fatality was associated with a lack of social support, a history of mental concerns, trauma, experiences of stigma, polysubstance use, and lack of coordinated healthcare [23]. The trauma inflicted through colonial violence in Canada has also been associated with overdose, for example, Indigenous women who had experienced child removal had over twice the odds of an unintended overdose than non-Indigenous women who had not lost custody [24]. It is vital to understand the intersecting structural conditions that PWUD face in seeking health and social supports.

The forces *external* to police that contribute to policing outcomes are part of these structural conditions. Studies show that adverse outcomes for people with mental health and substance use issues are largely due to social, political, and legal forces outside the control of police officers [12]. Examples of environmental factors include residential mobility, community socioeconomic conditions, social disorganization, and availability of alternative community resources [25]. Such factors influence police encounters among PWUD for sociostructural reasons (e.g. housing instability and drug use visibility) and socially disorganized areas will generally have higher levels of crime and police presence—lacking informal social control and social cohesion [26, 27]. Studies also show that socioeconomic factors play a critical role in the overdose epidemic; people who die from drug poisoning are significantly more likely to be unemployed and to be homeless or precariously housed [28].

Borrowing from research focused on police encounters with people with mental illness in the USA, Wood et al. [12, 29] note that police spend a lot of time brokering impermanent solutions to chronic structural vulnerability. In relation to responses to mental health crises, they suggest that we cannot define or understand police effectiveness without considering whole-of-system effectiveness, including an examination of the health, social, and criminal justice systems and their interrelatedness. Wood and colleagues present a framework for conceptualizing police work in the absence of robust responses to structural issues, such as homelessness, poverty, domestic violence, and unmet health care needs, including substance use disorders. According to the framework, which they call “principled encounters”, police should be expected to treat people with dignity, respect, and concern; to resolve encounters in ways that reduce immediate harms, avoid stigma, and promote public safety. However, improving long-term outcomes for individuals and their communities remains “contingent on the capacity of the wider system to do its job” [12]. Understanding how the health and social service systems’ capacity to meet the needs of the community has directly impacted policing—particularly among marginalized groups—has not been examined in depth in criminological or health research.

### Providing care and support for PWUD: a whole-of-system lens

Police encounters with PWUD occur at the intersection of criminal justice, public health, and social services. Police often feel compelled to perform health-related and social service roles for which they do not feel qualified, and they recognize negative consequences for both police agencies and the communities they serve [30]. In this study, we examine police officers’ everyday policing experiences interacting with and providing support to PWUD as well as their perceptions of how PWUD are affected by the different service systems, including the criminal justice, health, and social service sectors. With reference to mental health and substance use, it is perhaps unfitting to use the word “system”, which suggests an order of integration and accountability that does not currently exist in Canada [31]. Instead, there are a multitude of agencies operating in government silos, with people seeking care having to navigate a fragmented system built around a variety of actors responsible for different parts of the care pathway. When there is an absence of regular and meaningful communication between different parts of the system (both inter- and intra-agency), a “silo effect” can occur [32]. People with mental illness, PWUD, and people with complex health needs regularly interact with law enforcement, making the collaboration between law enforcement and health services critical. Unfortunately, there is often a disconnect between police contact and positive outcomes due to a variety of challenges including lack of training, resources, and collaborative support [33].

In this study, we use a systems theory lens to situate interactions between PWUD and police officers within the broader institutional and structural contexts in which they operate. Systems theory implies that all parts of an organization are interrelated and therefore a change in one area affects a change in another [34]. For example, deficits in emergency psychiatric service can lead to more people with mental illness being detained and arrested by police [35]. Coleman and Cotton [9] have used the systems orientation to examine relationships across sectors in the contemporary policing environment, explaining that the police system and mental health system are “sub-systems” within a larger social services system intended to benefit all citizens. Leischow and Milstein [36] have also explained that advances in public health require systems thinking and modelling, emphasizing a perspective that “considers connections among different components, plans for the implications of their interaction, and requires transdisciplinary thinking”. Meeting the needs of PWUD is a public health issue that necessitates examining the complex part-and-whole interactions [37] among the systems that respond to drug use. In this paper, we view police agencies as dynamic organizations that need to adapt to changing environments, rather than autonomous entities.

The aim of the current paper is to explore the positionality of police within the broader systems in which they operate (i.e. justice, health, social services), and how their interactions with/their ability to meet the needs of PWUD are shaped by this context. From the perspective of police, we present some of the key issues at the heart of the comprehensive failure to meet the needs of PWUD. The “total systems failure” refers broadly to the myriad ways that the justice, health, and social service systems fail, both separately and in combination, to meet the needs of PWUD.

## Methods

This study is part of *The Canadian Drug Laws Study*—a qualitative research project on drug enforcement experiences and views towards drug laws among police and people who use drugs. The current study focuses on qualitative data from semi-structured interviews conducted with Royal Canadian Mounted Police and municipal police officers across the province of British Columbia, Canada*.* The interviews were conducted privately over the telephone between July and September 2020. Each participant provided informed consent. The inclusion criteria were English-speaking officers with recent experience of drug enforcement or working with PWUD, and interest in speaking about these experiences. The study recruitment poster was shared to police officers through known networks as well as through police Chief’s identification of potentially eligible officers. Data collection occurred concurrent to data analysis to inform the purposeful sampling criteria [38, 39] ensuring that we captured a variety of perspectives from different genders, ethnicities, and regions, including rural and urban settings. Sampling and analysis progressed until no new relevant information emerged [40]. Data saturation occurred after fourteen interviews, at which point two more interviews were conducted to ensure comprehensiveness of the dataset.

The interviews were audio-recorded and later transcribed verbatim. Interview transcriptions were deidentified and stored on a secure cloud-based storage site located at Simon Fraser University (the Vault). Audio-recordings were permanently destroyed after transcription. Consent forms were provided to participants via email in advance of the interview and the key points were explained verbally by the interviewer. Consent was provided verbally prior to starting the interview recording. Interviews were performed using a semi-structured question guide developed by the researchers. The guide focused on four main areas of interest: (1) police officers’ experiences in enforcing simple possession and working with PWUD, (2) views on drug laws, alternative legal frameworks, and harm reduction, (3) perspectives on the policing role, and (4) policing experiences during the COVID-19 pandemic. The questions were informed by our research aims, previous experience conducting research with police officers, as well as a review of the relevant literature. As interviews and concurrent data analysis progressed, the wording of questions in the interview guide and probing questions evolved, providing an opportunity to deepen our understanding of topics and gather insights not initially considered. Examples of questions relevant to this current study include: “What do you see as your role as a police officer in terms of providing public health and social support to the community?”; “Do you have the necessary training and preparation to provide public health/social support services?”; “Do you think police should have a role in harm reduction?”; and “Do you think you’re able to make an impact in your position? What type of impact?” As the interviews unfolded, the interviewers wrote individual memos to record observations about each interview, salient ideas, and additional questions.

### Analytic approach

Specific questions about systems and the structural contexts of police work were not *explicitly* asked in the interview guide. Rather, the themes presented in this paper emerged inductively through officers’ descriptions and accounts of interacting with PWUD in their everyday work. Given that the interviews were focused on officers’ experiences of working with PWUD, participants regularly situated themselves within the larger context of services that are designed (in theory) to address the health and social needs of PWUD.

Transcripts and memos were imported into NVivo 12 [41] and any identifying information such as names and specific events were removed or anonymized. To further promote the anonymity of participants, no identifying information such as gender, ethnicity, or geographic region is tied to any participant quotes in this paper.

The method of analysis was a combined technique of inductive and deductive thematic analyses [42]. A preliminary coding framework was developed with input from all authors, aided by the reflexive work done throughout the study (e.g. memos, meeting notes, interview debriefs). This process involved a thorough reading and re-reading of the data to identify relevant patterns and organizing ideas [43]. Once the authors agreed on the coding framework, the initial codes were tested on a small subset of the dataset. The authors met as a group, and discrepancies in coding and interpretation were discussed and the data were revisited to ensure saturation and promote trustworthiness of the findings [38]. This researcher triangulation process [44] was used consistently throughout the study. After the first round of coding, the first author revisited the literature, codes, and memos, and then performed a second round with the goal of solidifying coherent relational patterns. It was at this stage of the analytic process and for the remainder of the analysis and write up that we applied the systems theory lens as an analytic tool, helping to situate police within a broader environmental and organizational context*.* We deliberately reviewed the data for dissenting or diverging viewpoints in our second round of coding. The authors used theory inductively and deductively to conceptualize and make sense of the experiences of the participants. Concurrent data collection and analysis was a key part of the interpretive analysis process and allowed for “ongoing engagement” with the data [45]. All authors met after the first author conducted two rounds of coding to synthesize and discuss the final themes presented in this study.

This study and methods described were approved by Simon Fraser University’s Office of Research Ethics (#2020s0231).

## Findings

### Participant profiles

Sixteen police officers participated in the study; nine (56.3%) were from the Royal Canadian Mounted Police and seven (43.8%) were from municipal police forces. Participants were an average of 43 years old (range 28–53 years) and self-identified mainly as male (*n* = 15; 93.8%) and white ethnicity (*n* = 13; 81.3%), while three (18.7%) participants identified as ethnic minorities. The median time of working as a police officer was thirteen years (range 8–13 years) and working within their current jurisdiction was eleven years (range 3–31 years). These participant characteristics can be found in Table 1.

**Table 1:** Participant characteristics (*n* = 16)

Participant characteristic	*n*(%)/median(range)
*Gender n (%)*	
Male	15 (93.8%)
Female	1 (6.3%)
*Ethnicity n (%)*	
White	13 (81.3%)
Indigenous	1 (6.3%)
Asian	2 (12.5%)
*Police force n (%)*	
RCMP	9 (56.3%)
Municipal police	7 (43.8%)
Time working as a police officer, in years Median (range)	13 (8–31 years)
Time as officer in jurisdiction, in years Median (range)	11 (3–31 years)
Age, in years Median (range)	46.5 (28–53 years)

### Qualitative findings

The findings presented below are organized into three overarching themes. The first two themes examine distinct but related systems-level challenges, including: (1) an ineffectual criminal justice system and (2) inadequacies in the health and social service systems. A third theme, “thoughts on systems reform”, focuses on collaboration across service sectors, including police officer perspectives on barriers and benefits to meaningful health and justice collaboration as well as suggestions for how to improve. A list of themes and sub-themes is provided in Table 2.

1. **“It’s a joke”: an ineffectual criminal justice system**

**Table 2:** Themes and sub-themes

Theme	Sub-theme
1. “It’s a joke”: an ineffectual criminal justice system	Failure to deter crime Failure to address drug use and the needs of PWUD
2. The “bottom of a huge societal funnel”: inadequacies in the health and social service systems	Lack of treatment and support to address structural vulnerability of PWUD “Dumped on the foot of the police”: defeat and demoralization among officers
3. “The system needs a radical change”: thoughts on systems reform	Benefits and barriers to intersectoral collaboration Systems reform and role redesign

From the perspective of police officers, the criminal justice system was one component of a wider network of systems that collectively failed to meet the needs of PWUD. For example, one officer, when explicitly asked about the justice system in Canada, replied: “*It’s a joke*”, speaking to their lack of confidence in its effectiveness. This sentiment was reflected in officers’ comments on the failures of the justice system in deterring crime and addressing drug use or supporting PWUD more broadly. For example, officers witnessed enforcement measures that perpetuated harms for people already suffering. The impact was that officers themselves felt ineffective as agents of change. Two sub-themes—failure to deter crime and failure to address drug use and the needs of PWUD—are examined below.

1.1. **Failure to deter crime**

Officers reflected on their everyday work in the community, often referring to the failures of the justice system in preventing crime. Officers described witnessing individuals repeatedly caught up in the justice system, reflecting its cyclical nature. The officers frequently referred to the justice system’s “revolving door”, with one officer noting, “I could arrest them one hundred times and I don’t think that will make any difference”. Part of the cycle that officers witnessed was due to individual’s contact with the justice system itself by creating more, rather than less, contact with criminal networks. In some cases, officers framed the criminal justice system as an opportunity for people to become further embedded in criminal networks. As an officer explained:

*I look at throwing individuals that have committed crime into jails, in the sense that they’re doing …minor property crime to fuel their drug habits… it’s a waste of the system. They don’t get clean in prison, I know that. They get drugs easily in prison. And they become embedded to the gangs that control that prisons as they’ve told us. And then they’re doing more work when they come out. Some of them become dealers themselves so the system needs a radical change.*

Officers saw correctional facilities as unsupportive places for PWUD that *further entrenched them in the drug trade*. From the perspective of these officers, the criminal justice system failed to promote positive health outcomes or trajectories for PWUD.

1.2. **Failure to address drug use and the needs of PWUD**

Officers also questioned the effectiveness of the criminal justice system addressing the *issue of drug use*. One officer said: “I think we’re getting a failing grade on how we deal with addictions in BC, and Canada, and we definitely need to be doing better”. Officers pointed to a lack of supports available through the justice system to address or prevent drug use itself, which was reinforced by a lack of supports available in the community. For example, officers observed that upon release from prison, people were provided with *no re-entry supports* and commonly returned to drug use:

*Somebody using drugs who is addicted, you arrest them you send them to court, the case goes through the court system. They’re back on the street they’re using again. It’s not working, it’s not doing anything. So, that’s frustrating. As an enforcement officer on the other hand, I do understand that alcohol and drug addiction is a health concern and health issues, I just wish there was more from the health side of things to help with this problem.*

Officers were cognizant of the justice system having little influence on deterring or preventing drug use. Considering the potential harms from incarceration, they questioned the effectiveness of the criminalization of drugs in terms of reduced harms or improved public safety. Notably, officers questioned the framing of drugs as a criminal issue rather than health issue. One officer said:

*When you used to deal with a person that was an alcoholic and they were on conditions to not consume alcohol, well, they’re an alcoholic and it’s not going to work. It’s designed for them to fail … If everybody else says that this is a mental health and health issue because they’re addicted, which I agree, the court system needs to recognize that too.*

Officers recognized that processing PWUD through the criminal justice systems was both ineffective and inappropriate for addressing drug use itself or the root causes of drug use. There was broad agreement that drug use would be better addressed through alternative systems.

2. **The “bottom of a huge societal funnel”: health and social service systems failures**
2.1. **Lack of treatment and support to address structural vulnerability among PWUD**

In addition to the shortcomings of the criminal justice system, officers reflected on issues in the health and social service systems. Failures in these systems were particularly evident when officers reflected on their history of navigating and accessing health services including treatment for PWUD. One officer explained:

*I’ve got too many examples where somebody says they want to get into treatment, and I’m calling [health services] on their behalf. And I’m waiting on the phone for 45 min… then the next thing you’re dealing with is you gotta figure out how you’re going to get them to detox. You know, that in itself is a whole pickle as well, especially now with policing. Because people in our management say that if you’re doing a voluntary transport of somebody in a police car, then there is potential liability… and all I want to do is get somebody help—they say they want help they want to get into treatment, and all I’m looking to do is get them into treatment… I’ve started to get relationships with [health services]… where I know some of the people. Where I can call them up and I can walk the person over… but I’ll be honest with you those didn’t really work well either.*

Officers’ experiences of connecting PWUD to treatment services reflected the high-level barriers and lack of accessibility to such services in the health system. It also reflected an informal triage role that officers played in the community. Due to the inadequacy of the health system and lack of formal integration with policing, the success of police “triage” may largely depend on individual police officers’ unique community relationships and networks.

Several officers pointed to other *structural and social determinants of health* (e.g. housing stability, childhood trauma) and their relationship to drug use. Some spoke about the repeated attempts of individuals trying to access social supports, such as housing, over years or decades without success and the harms experienced from these inequities. One officer commented on the ubiquity of exposure to the foster care system among the people they see on the street: “I don’t know if I can recall any kids who were on our street, you know, in the teenage years, that weren’t in the foster system”. This quote and others echoed instances of people experiencing early-life trauma that contributed to their drug use and chronic socioeconomic deprivation. Officers also often reflected in their own failed efforts to broker social and health supports including housing and treatment options for PWUD. For instance, one officer spoke about his experience of working with people with mental illness and witnessing the total systems failure: “there’s nothing in place to help them. There’s absolutely nothing… complete abdication from all three levels of government to care for people properly”.

Importantly, the multiple, overlapping disparities that PWUD experienced highlights the need for a full spectrum of services to address the intersectional structural vulnerability of PWUD. However, officers also acknowledged the complexity of addressing this challenge:

*People suffering from trauma, great you get them to stop using drugs, then what? A lot of them, if it [the trauma] happened when they were young, they dropped out of school, they have no education, they have no marketable skills to get a job. So, great, they stopped using drugs, what are they going to do?... What are we doing to train them in, to give them a career, to give them a skill? We’re not doing that either, right? And that seems to be turned into, ‘well that’s getting too involved in somebody’s personal life’… How about presenting it as offering people an opportunity? … I think it [service goals] has to be a lot more comprehensive than just simply getting people off drugs.*

In the context of working at the intersection of inadequate justice, health, and social systems, officers were acutely aware that these systems were failing to comprehensively address the structural determinants of health. They highlighted that in many cases, *structural vulnerability* among PWUD, rather than drug use itself, was what drove people into criminal justice contact. Many commented on the increased likelihood of working with “people who are basically street entrenched or generally vulnerable populations or have an unstable housing situation”. This structural vulnerability of homelessness was captured by one officer who said:

*What percentage of this population lives in their own homes and uses [drugs] and never comes across my vision? It’s fairly large. But generally, my interaction with substance use is usually at the higher end of crisis. Heavy addiction and mental health, and homeless.*

Overall, officers recognized that individuals’ health, social, and justice outcomes differed based on socioeconomic status. They also commented on the role of poverty and criminalization, as well as access to treatment: “There truly is a two-tier medical system in Canada. Because the wealthy can go to treatment, and the poor people get harm reduction and a waiting list”. Officers openly acknowledged the complexity of the health and social issues facing PWUD, calling for a comprehensive, whole-of-system response.

2.2. **“Dumped on the foot of the police”: defeat and demoralization among officers**

Officers often felt that they were at the end of a long line of systems failures experienced by PWUD. They felt compelled to pick up the proverbial pieces of these failures, feeling responsible to help, support, and meet the needs of PWUD. One officer explained:

*A lot of people who are down there [low-income location] they have been failed… by every social agency, that exists under the government. And now they have been left for the police to deal with. They’ve been failed in the educational system, the social assistance system, the welfare system, the medical system, and when it all falls through the cracks it gets dumped on the foot of the police to deal with.*

However, officers felt ill-equipped to provide drug- and other health-related supports such as triaging, particularly for officers who lacked training or experience working within these systems. Some officers recognized the harms of relying on untrained, ill-equipped officers to respond to health and drug-related issues. As a result, there was a sense of hopelessness, cynicism, disappointment, and despair among officers in these roles. These feelings are captured among one participant who said:

*I’m absolutely disgusted by the fact that there is a lack opportunity for people to get themselves clean. [Sigh] I’ve worked on it for 16 years and I’m still dealing with people that I’ve met as kids... there is no opportunity for them to get out, and I’m so, so, saddened… disappointed about that.*

Some felt frustrated by the reliance on officers to navigate inadequate systems and their functional role within them. Officers questioned their own effectiveness at the intersection of these systems, and recognized that PWUD themselves were negatively impacted by the total systems failure:

*There is no one else. There is no one*—*there is a huge gap that no one else fills, and… that is sort of the bane of the existence of the police. We see ourselves at the very bottom of a ****huge societal funnel****, whenever there is an issue that no one else can deal with.*

Participants’ reflections of working with PWUD offered a sense of *cynicism and demoralization* from repeatedly observing criminal justice system failures and the subsequent harms to PWUD. Officers also questioned their own role in perpetuating an ineffective system and expressed a sense of being demoralized and defeated. Officers “see the same people over and over again” and explained that they “don’t feel like [they’re] helping anyone”. Officers questioned their role in this system and often recognized that they were contributing to the problem rather than offering any meaningful solutions.

3. **“The system needs a radical change”: officer challenges and suggestions for systems reform**
3.1. **Benefits and barriers to intersectoral collaboration**

As illustrated in Themes 1 and 2, officers were concerned about the shortcomings of the justice, social, and health systems. They acknowledged the need to bolster collaboration between providers and coordination of efforts across these systems to comprehensively address the health and social inequities PWUD experience. Officers offered several suggestions for how the system could be reformed. One suggestion was to promote effective collaboration through co-response models that already existed, such as mental health cars that paired police officers with a psychiatric nurse. Officers highly regarded these collaborative programs and felt that more were needed:

*We had a psychiatric nurse working side-by-side, and actually paired up with a police officer on their shifts… way more effective to have a person that’s got experience and training in that area to be there to deal with the mental illness portion or the substance abuse problem in the realm of the safety of having an officer there. So, that’s definitely a good partnership to have, and its good to have that resource.*

Officers respected the expertise and skills of mental health professionals and wanted more and improved access to their support. One officer remarked “we need to be more collaborative with health services, to get people with the proper training out there to assist us”. The collaboration was seen as more effective than increasing police training in mental health and substance use: “police are woefully inadequate for those positions”; “a registered psychiatric nurse, they’ve gone through their nursing, they’ve done years in the community, they have all the resources for this”. However, officers concurrently believed that both officers and health professionals should not operate in isolation or silos, but rather they could collaborate and coordinate their efforts. One officer suggested:

*I think that a better partnership should be set up … and I love working with social workers, and mental health workers. I think they’re great, they’re the people who are trained to do those things, but it doesn’t need to be siloed…police can work along side these people to get the benefits that we need, right.*

There was overall support for having trained mental health professionals, including nurses and mental health liaisons, work in close collaboration with police.

Alongside the common desire for more collaboration between actors in the health and social systems and police, officers noted several challenges and reasons why systems remained siloed. Officers indicated an overarching lack of communication and understanding of the roles and responsibilities across sectors. Some officers admitted that tension stemmed from a lack of understanding and appreciation for the mandate of one another. One officer who said:

*There's no communication. And apparently, they've had it [health service for PWUD] for six months that they actually have a nurse on site that prescribes suboxone, I think it is, to help them with their addictions. I had no idea up until last week. It’s been going on for quite a while, I drive by there 100 times a day. That should have been knowledge passed on to the police, that there’s a place where we can send people who have an addiction. There’s zero satisfaction for me arresting a guy every day over and over, how can someone get satisfaction out of that?*

This officer felt frustrated that police were not informed of new health services, contributing to the difficulties faced in connecting PWUD to such services. In situations where there was overlapping health, social, and/or public safety concerns, officers often found themselves in a “blame-game”: “there’s a lot of ‘this is your problem’ and finger-pointing”, speaking to the inefficiencies of the system. One officer explained:

*You’ll get some cops that’ll think, “oh this isn’t my problem, this is a health problem, you’ve heard it in the news, drug use is not my problem, this is yours.” And the nurse will be like, “well, [the client] seems violent, how am I supposed to deal with this right now in the ER?”… I’ll hear it from both sides. I think there needs to be cooperation and the walls need to come down.*

Disagreements about who is ultimately responsible (health services or police) in any given situation leads to system inefficiencies and ultimately, poor outcomes for clients. Some talked about how privacy issues also limited effective communication between police and health services:

*If you view substance use from the lens that it’s a healthcare problem, then healthcare has generally extremely private information... So, it makes it a lot harder to have conversations back and forth between health agencies and, you know, the other realms that substance use branches into, like housing and policing, to have these open and frank conversations.*

For officers, the lack of information shared between health and social services and themselves was a barrier to them effectively providing support for PWUD and other marginalized groups. The limited communication reinforced the silos or “walls” between the justice system and other systems. As one officer explained:

*Now everybody shields themselves with the privacy and we don’t share as much together. So, I know we get a lot more friction from [health authority name] because of their policies now, where it used to be we were more forthcoming with each other … I find there are a lot more walls between everybody. And it’s like we’re in the interest of the patient or client or whatever, but now we’re just gonna play the privacy card, ‘I can’t share anything’ … so I think there’s a lot of bureaucracy going on.*

Officers felt that such privacy concerns were counterproductive to collaboration, but they did not seem to acknowledge the potential criminalization of these individuals if their privacy was breached.

Not all officers felt disconnected from the health and social systems, but it took active efforts to develop collaborations and cooperation in them. Some explained that their units developed *strong relationships* with community service provider. However, they also felt that developing these relationships did not come easily—it took time, effort, and openness: “we work very hard to cultivate those relationships”; “it took time to build the relationship—three and a half years”; and “there’s always going to be people on both sides who don’t really enjoy the other person's point of view, but we had enough common ground that we were able to develop some guidelines that worked well for everybody”. The need to build relationships over time underscored the perceived lack of trust that service providers had towards police officers. As such, it was necessary to proactively build relationships with health and social services to build trust and cooperation.

*I let the staff know, and the harm reduction people, and who ever came in the building that I was there just as much for them as I was for the client. And just over time they started to be able to rely on me and trust me and know I was there for the best interest for the client as well as the people working there. So, I think just my approach and my genuineness and just time, and patience and even to today I still get calls from them. I’m not even working that job anymore and they still call me on my phone, leave a message—‘hey we really need you’.*

Officers valued structured opportunities for discussion and relationship-building. They recognized meaningful and positive collaboration with community service providers as critical to improving care.

3.2. *Systems reform and role redesign*

While none of the officers claimed to have the ultimate solution, many emphasized that *systems changes* were required to meet the needs of PWUD and improve outcomes. One officer suggested redistributing and connecting efforts across the systems:

*It’s all intermixed and it should be that there’s a nurse and a social worker and a mental health worker working in the police department. And vice versa, you can have the police officer in the hospital if they need one.*

For this officer, it was essential to recognize the intersection of the social, health, and justice systems to promote collaboration and cooperation between actors within them. Another officer took this concept further by suggesting police officers formally position themselves in terms of health and social support. This officer suggested creating a new class of policing focused on mental health support:

*There needs to be a new kind of police officer…. the one that’s in charge [during mental health call] and has a better chance of talking to someone with mental health… Could follow the same thing in harm reduction… having a kind of a social worker class of police officer, or mental health police officer, something new.*

This reimagining of the policing role stemmed from a clear recognition that the justice, health, and social systems, when acting in isolation, were failing to comprehensively support and address the needs of the community. This quote also speaks to the recognition that treating health and social crises solely as a justice issue was inadequate and inappropriate. Rather, officers recognized that many of the situations they dealt with were fundamentally social or health issues. Instead of addressing them through “one pillar”, such as healthcare alone, partnerships across systems were needed:

*[Drug use]… is a complex problem. Often related to mental health, homelessness, poverty, things like that. I think if we look at it just as one…like one pillar is going solve the problem, we’re looking at it the wrong way.*

Officers did not see themselves being fully removed from issues at the intersection of justice and public health, even in a reimagined/reformed system. There was a general assumption that police will continue to interact with people with complex health and social needs, particularly in the face of the ongoing criminalization of drugs, as part of their day-to-day work. However, they identified the need for more specialized liaison roles that could work across health and policing, as well as increased formalized relationships across sectors, to improve the health and social trajectories of people they encounter.

## Discussion

This study has presented findings of how, from the perspective of police, the justice, health, and social systems have collectively and comprehensively failed to meet the needs of PWUD. Officers viewed the criminal justice system as an ineffective response to drug use, cycling PWUD through a “revolving door” that led to negative health and social outcomes. These failures were coupled with glaring gaps in social and health service systems which inadequately positioned police officers as frontline responders to the complex needs of PWUD. Officers recognized that PWUD ultimately suffered from the structural impotence of this “total systems failure”. As actors trying to respond to the needs of the community, police officers feel defeated and demoralized by the ineffectiveness of these systems to reduce crime or support PWUD. These findings have major implications in terms of health, social, and justice system reforms.

For a host of reasons (including a shortage of investment in health and social services, police being uniquely available 24 h per day), police have become the public service tasked with response to a range of crises including those related to PWUD. Our study is consistent with others that have highlighted the inefficiencies of using the criminal justice system to prosecute drug-related crime from the perspective of police [29, 46, 47]. Law enforcement internationally has expressed frustration about the revolving door phenomenon—i.e. PWUD cycling in and out of prison at considerable individual and community-level cost [47, 48]. However, police officers and community members do not agree on the extent to which police should be formally involved in health crises and harm reduction. For example, a paper from *The Canadian Drug Laws Study,* which examined role tension within policing, found that some officers felt that police should be resourced and trained to respond to health issues whereas others felt this was an inappropriate function of police, and one which they have been compelled to take on by necessity [46]. There are conflicting and yet strong calls for both the inclusion and exclusion of police from health systems. Beyrer argues that the role of police can be “re-envisioned” to include harm reduction and by doing so, the goals of both disease prevention and public safety can be met [49]. Along the same lines, del Pozo et al. [50] stated that police discretion should be guided by a public health ethic that operationalizes public safety with “decisions that equitably improve health outcomes” and implementing this ethic can precede formal policy change [50]. Validating the role of police in reducing the harms associated with low level crime has been termed *harm reduction policing*—a form of policing that “seeks to build the capacity of systems to address health needs while validating the police mission to protect public and individual safety”[48].

In contrast, some scholars that have examined policing within specific harm reduction contexts [for example, operating safe consumption sites (SCS)] have argued that police should remain excluded from the health sphere because the disciplinary conditions created by police, often under the guise of responding to social disorder, undermine public health efforts and foster distrust of police among PWUD [51]. A recent study of policing boundaries for an SCS found that from the perspective of service users and SCS staff, police should not be present at the SCS as they are a deterrent to service uptake [52]. If police are going to be in the vicinity of an SCS, the expectation is they would be knowledgeable about the service, willing to refer, and supportive of harm reduction [52]. Interestingly, there were mixed opinions in this study about whether and to what extent police should receive overdose response training. The debate about the role of police in responding to PWUD is ongoing and rapidly evolving, particularly in light of the increasing death toll associated with illicit drug supply toxicity.

Our study contributes to the broader literature that investigates the overlap between justice and health with respect to PWUD, but it goes beyond what is currently available by examining the police positionality within that overlap, and the perceived implications for meeting the needs of PWUD. A main contribution of this study is evidence that police are continuing to serve as de facto frontline responders to mental health and substance use-related crises in their everyday work in BC. As noted, officers were often not trained nor appropriately or adequately positioned to broker health and social services for PWUD and other marginalized populations. Rather, they took up these roles by default, underscoring the importance of considering police as a “sub-system” within a broader system of care, and one that is directly impacted by deficits in the other sub-systems. In the face of multiple system failures, police will inevitably continue to play this role. This finding is concerning for a few reasons. First, while police are being asked to “stand down” [16], there are glaring social and health service gaps needing investment and expansion. Shifting mental health and substance use supports away from police requires “an adequately funded and functional community mental health system with a workforce ready to embrace its role in crisis response” [53]. A clear implication is that police reform deliberations cannot occur in isolation—they must consider the context in which police work and the interrelatedness of policing with other systems, including the other levels of the justice system.

With slow-acting governments, a lack of reforms, and inadequate resources contributing to a “total systems failure”, it is important to recognize that the positioning and role of police officers in the community will persist. It has been suggested that police officers should receive *more* training and funding to promote social justice and equity [9, 54]. Alternatively, others have argued that there needs to be a *disinvestment* of policing to lessen the role of police in the community, coupled with larger structural changes such as legal reform [55]. Both approaches will require a systems perspective to understand how the health, social, and justice systems overlap. The findings presented in our paper underscore the importance of taking this perspective.

Despite widespread recognition of substance use as a public health issue, the possession of illicit substances remains criminalized, and the result is a problematic misalignment between police and health service agencies [56]. As such, separating police from health and social systems may require addressing the criminalization of drug use altogether. Officers themselves saw drug use as a health and social issue—not a criminal one—and noted the ineffectiveness and harms produced from the criminalization of drugs. However, in the context of a shifting drug policy context where the decriminalization of drug possession is under consideration, as in British Columbia, the role and position of drug enforcement are in flux. For instance, police diversion models are being considered as alternative, non-criminal responses to drugs [57]. Diversion models hinge on police in triage roles; therefore, their collaboration and coordination with other systems will be required. Alternatively, full decriminalization has the potential to decouple policing from these systems altogether. However, even if police diversion and decriminalization models are implemented, police officers may continue to respond to the needs of PWUD due to the inequities they face and the failures of health and social systems. Again, investing in and reforming systems *beyond* the criminal justice system should be a necessary step. Alternatively, the shortcomings of these systems may undermine reforms altogether.

While decriminalization and decoupling policing from the health and social systems is one approach to addressing the structural violence PWUD experience, it does not address the *intersectional* vulnerabilities faced by many PWUD. Our study points to the structural failings of the health, social, and justice systems that impact people experiencing poverty, mental health issues, housing instability, unemployment, racism, and other social inequities. These inequities will still exist in the face of reforms, such as drug decriminalization and policing reforms, and may significantly contribute to criminal justice-related outcomes for PWUD experiencing these intersectional structural vulnerabilities [19]. For instance, PWUD who are also homeless experience significantly more frequent police encounters than those who are housed [13]—this issue will not be addressed through drug decriminalization alone. As Freidman explains [58], calls made to police are “driven by real problems, that policing exacerbates, but for which there often is no alternative present… we won’t ameliorate the harms caused or exacerbated by policing unless we get at the underlying problems themselves”. Addressing the total systems failure will involve an examination of the shortcomings across systems and will likely require substantial funding and reforms. Future research is needed that can further untangle the main structural shortcomings and alternative frameworks to address the intersectional structural vulnerability that many PWUD experience.

### Limitations

This work fills a critical gap in the Canadian literature, gathering the perspectives of police officers on drug policy and interactions with PWUD. There are a few notable limitations. First, although we attempted to purposefully sample a variety of views and experiences among officers, the study itself may have attracted officers with more lenient practices or liberal views. As well, collecting data over the telephone may have limited the rapport between the participant and interviewer. The findings do not necessarily represent the views and experiences of all police officers in BC. Views from other police services in Canada or from those most affected by drug enforcement—PWUD themselves—may provide different insights on this topic. We found that views and experiences did not differ considerably across the province of BC. This study employed an inductive approach, and so it is theoretically possible that if we were to ask explicit questions related to the themes described, that we would find more varied sentiments. This paper was exploratory in nature and was not designed to study differences based on the police officers’ organization (municipal vs. RCMP), rank, or location. Future research should explore how/if organizational and geographic factors impact police partnerships and interactions with the health and social service sectors. It is essential to ensure that future policies are sensitive and responsive to the lived experiences of PWUD. The perspectives of employees of other sectors, including health and social services, should also be considered when re-conceptualizing the appropriate role for police in health-related crises, as well as new roles that should be created to support the goals of improved outcomes and reducing recidivism.

## Conclusions

Sparked by the murder of George Floyd by police office Derek Chauvin in the USA, advocacy groups in jurisdictions all over the world are putting new pressure on legislators and policymakers to “defund the police”. The growing “defund” movement has made calls ranging from cuts to police budgets to total abolition of police forces [59]. Tough on crime political ideology, the war on drugs, and neoliberalist policy agendas that have resulted in significant cuts to health and social services have together led to significant unmet mental health and substance use needs in the community alongside increased social deprivation over the last several decades. In turn, police have become responders-by-default for issues that are fundamentally related to people’s health conditions and chronic structural vulnerability.

Issues at the health and justice intersection are driven not only by individual-level behavioural and health conditions but by social and economic forces at the systems level [60]. Meares et al. ([61], para. 10) argue that “narrow conversations about ‘defunding’ assume that the other [non-police] parts of the system are operating the way they should be, and we know they’re not”. While this paper centres the views of police, it is important that mental health and social services become key partners and advocates, calling for policies and resources that are needed *across* systems to lessen the involvement of police in drug- and behavioural health-related issues, and most importantly, to improve outcomes for structurally vulnerable members of our communities.

## Data Availability

Data sharing is not applicable to this article as no datasets were generated. The identifiable qualitative data from the interviews will not be shared outside of the research team as per our consent declaration.
